# Short-Term Effects of NO_2_ Exposure on Hospitalization for Chronic Kidney Disease

**DOI:** 10.3390/toxics12120898

**Published:** 2024-12-11

**Authors:** Wancheng Zhang, Ye Ruan, Jianglong Ling

**Affiliations:** 1 Longyan First Affiliated Hospital of Fujian Medical University, Longyan 364000, China; 2 School of Public Health, Lanzhou University, Lanzhou 730000, China; 3 Medical Center for Neck and Low Back Pain, Xijing Hospital, Fourth Military Medical University, Xi’an 710000, China

**Keywords:** NO_2_, exposure, chronic kidney disease, hospitalization

## Abstract

This study investigates the correlation between short-term exposure to nitrogen dioxide (NO_2_) and hospitalization for chronic kidney disease (CKD) in Lanzhou, China. A distributed lag nonlinear model (DLNM) was employed to examine the relationship between changes in NO_2_ concentration and CKD hospitalizations. Subgroup analyses were conducted to assess the sensitivity of different populations to NO_2_ exposure. A total of 35,857 CKD hospitalizations occurred from 1 January 2014 to 31 December 2019. The average daily concentration of NO_2_ was 47.33 ± 17.27 µg/m^3^. A significant exposure response relationship was observed between changes in NO_2_ concentration and the relative risk (RR) of CKD hospitalization. At lag0 (the same day) and lag0-1 (cumulative same day and the previous 1 day) to lag0-4 (cumulative same day and the previous 4 days), NO_2_ exhibited a harmful effect on CKD hospitalizations, with the maximum effect occurring at lag0-1. For every 10 µg/m^3^ increase in NO_2_ concentration, the RR of CKD hospitalization was 1.034 [95% confidence interval (CI): 1.017, 1.050]. Subgroup analyses revealed that the adverse effects of NO_2_ were more pronounced in females and individuals aged ≥65 years. The harmful effects were also more significant during the cold season. In conclusion, short-term NO_2_ exposure is associated with an increased relative risk of CKD hospitalization. Continuous efforts to improve air quality are essential to protect public health.

## 1. Introduction

Chronic kidney disease (CKD) is defined as a condition characterized by persistent abnormalities in urine composition, structural renal abnormalities, or impaired renal excretory function, ultimately resulting in the loss of functional renal units [1]. CKD is a progressive and irreversible condition [2]. In the early stages, CKD is often asymptomatic, leading many patients to be diagnosed only when the disease has progressed to a more advanced stage. As the condition progresses, it can result in a significant decline in kidney function, and it may eventually progress to end-stage renal disease.

It has been reported that CKD affects 750 million people worldwide [3]. From 1990 to 2019, the global number of CKD-related deaths increased from 1.57 million to 3.16 million, while the number of cases rose from 7.8 million to 18.99 million, with the mortality rate rising by 41.5% and the prevalence increasing by 29.3% [4,5,6]. CKD is currently the third leading cause of death worldwide in terms of growth rate [7]. The situation of CKD in China is particularly concerning, with its prevalence increasing from 6.7% in 1990 to 10.6% in 2019, and the number of affected individuals reaching 150 million by 2019 [8]. In 2016, the treatment and management costs for CKD patients in China amounted to RMB 27.6 billion [9]. CKD has emerged as a significant global public health challenge, with the number of affected individuals continuing to rise and the treatment burden escalating.

The etiology of CKD is complex and multifactorial, closely related to a variety of factors. Common risk factors include the long-term use of anti-inflammatory drugs, hypertension, diabetes, and autoimmune diseases [10,11]. Lifestyle factors also play a crucial role in the development of CKD [12]. Unhealthy lifestyles, including sedentary behavior, an unbalanced diet (e.g., high salt and high fat intake), and smoking, significantly increase the risk of kidney disease. Additionally, environmental factors also play a critical role in the onset and progression of CKD [13]. Moreover, heavy metals (e.g., lead and mercury) and chemicals (e.g., phthalates and melamine) are nephrotoxic, and their accumulation can cause kidney damage, thereby increasing the risk of CKD [14]. Air pollutants are also a significant risk factor for CKD. Studies have shown that air pollution adversely affects kidney health and may accelerate the progression of kidney function decline [15].

Air pollution presents substantial health risks, disease burdens, and economic losses. Over 91% of the global population resides in areas where air quality exceeds the concentration limits set by the World Health Organization (WHO) [16]. Air pollution not only contributes to respiratory and cardiovascular diseases but also impacts the nervous and immune systems [17,18]. About 4.2 million people worldwide die each year from air pollution exposure, according to a survey of the WHO, and more than 1.24 million deaths are attributed to air pollution in China [19]. Air pollution has emerged as a major public health issue worldwide.

Previous studies have investigated the impact of air pollutants on CKD [20,21,22,23,24]. However, due to differences in population characteristics, economic conditions, geography, and climate, the findings often vary across different regions. Therefore, conducting research in diverse geographical settings is essential to gain a comprehensive understanding of the local context. At present, no studies have specifically examined the effects of air pollution on CKD hospitalizations in Lanzhou, China. Lanzhou, located in the northwest inland of China, is a typical river valley city with unique climatic conditions. The region’s distinct geography and climate contribute to significant air pollution. As such, conducting research in Lanzhou is of considerable importance to elucidate the local situation and its potential health impacts.

## 2. Materials and Methods

### 2.1. Study Area

Lanzhou (E 102°36′–104°35′, N 35°34′–37°00′) is the capital of Gansu Province, located in the inland region of northwest China. As a significant transportation hub and industrial base, Lanzhou is traversed by the Yellow River. As of 2021, the city has a resident population of approximately 4.36 million, and it covers an area of about 13,100 square kilometers. The city’s four major urban districts—Chengguan, Anning, Qilihe, and Xigu—are densely populated and situated on the plains formed by the Yellow River, with these areas distributed along both banks of the river. Surrounded by mountains, Lanzhou is a typical valley city. This study focuses primarily on these four urban areas.

### 2.2. Data Collection

CKD is a prevalent, chronic, non-communicable disease, with its diagnosis and treatment spanning various levels of healthcare institutions. In selecting study sites, we considered factors such as hospital tier, service quality, the completeness of inpatient data, geographic location, and departmental structure. We focused on 21 public hospitals at the second tier or above, located in the four main districts of Lanzhou. These hospitals are well equipped, featuring advanced electronic medical record systems and cutting-edge diagnostic and treatment technologies, enabling them to provide high-quality healthcare services that meet the needs of the majority of local residents.

For this study, daily inpatient data were collected from these hospitals over the period of 1 January 2014 to 31 December 2019. The data included information on patients’ gender, age, admission diagnosis, date of admission, and International Classification of Diseases, 10th Edition (ICD-10) codes. Specifically, we extracted data for patients diagnosed with CKD (ICD-10: N00–N19). Patient records with incomplete or incorrect information (e.g., missing or incorrect gender or age) were excluded. This study was retrospective in nature, with data collected for administrative purposes, and as such, it was exempt from ethical review.

Air quality data for the same period were obtained from air quality monitoring stations in Lanzhou. These data consist of hourly concentrations of air pollutants recorded via four national air quality monitoring stations located within the city: the Biological Products Institute, the Railway Design Institute, the Lan Refining Hotel, and the Provincial Staff Hospital. Lanzhou is a river-valley city with a narrow east west orientation and a constrained north south span, and its residential areas are primarily concentrated along the city’s main roads. The air quality monitoring stations are situated up to 15 km from residential areas, ensuring that the data accurately reflect the exposure levels of local residents [25]. The data primarily consisted of daily average concentrations of fine particulate matter (PM_2.5_), respirable particulate matter (PM_10_), nitrogen dioxide (NO_2_), sulfur dioxide (SO_2_), carbon monoxide (CO), and the maximum 8-h ozone concentration (O_3_ 8 h). Meteorological data for the same period were sourced from the China Meteorological Data Service Network, including daily average temperatures and daily average relative humidity. These data were collected and analyzed by trained professionals, ensuring their completeness and high quality through rigorous quality control procedures.

### 2.3. Statistical Analysis

Firstly, a descriptive statistical analysis was conducted on CKD hospitalization, air pollutants, and meteorological factors to reveal their general characteristics. The primary statistical indicators used included the mean, standard deviation (SD), minimum (min), 25th percentile, median, 75th percentile, and maximum (max). Spearman’s rank correlation method was applied to examine the relationships between air pollutants and meteorological factors.

Secondly, a distributed lag nonlinear model (DLNM) was employed to examine the relationship between NO_2_ and CKD hospitalization rates. The adverse effects of air pollutants on disease hospitalization are persistent and lagged. Furthermore, the daily number of hospitalizations due to CKD is low relative to the total population, and its distribution can be approximated by a quasi-Poisson distribution. To address potential overdispersion, the quasi-Poisson distribution was used as the link function. We constructed a single pollutant model to investigate the impact of NO_2_ exposure on hospitalizations for CKD. Day-of-week and holiday effects were included in the model as categorical variables to account for potential confounding. Temperature and relative humidity were also incorporated, with their effects controlled using natural cubic spline (ns) functions. Similarly, time variables were modeled using ns to adjust for long-term trends and seasonality. Based on prior relevant experience and the Akamatsu Information Criterion, the degrees of freedom (df) for the temperature, relative humidity, and time variables were determined to be 4, 4, and 7, respectively. We constructed a DLNM to estimate the relationship between changes in NO_2_ concentration and CKD hospitalizations, combining a generalized additive model (GAM) with a quasi-Poisson distribution. The single pollutant model for NO_2_ developed in this study is as follows:

$$\mathrm{Log}(E(Y_{t}))=\alpha +\mathrm{cb}({\mathrm{NO}}_{2},\;\mathrm{df}=4)+\mathrm{ns}(\mathrm{Temperature},\;\mathrm{df}=4)+\mathrm{ns}(\mathrm{Relative}\;\mathrm{humidity},\;\mathrm{df}=4)+\mathrm{ns}(\mathrm{Time},\;\mathrm{df}=7*\mathrm{year})+\mathrm{Dow}+\mathrm{Holiday}$$

where $Y_{t}$ is the number of hospitalizations for CKD on day *t*. $E(Y_{t})$ denotes the expected number of hospitalizations. α denotes the intercept. *t* denotes the date of hospitalization. cb() denotes the two-dimensional cross-base function of NO_2_. df denotes degrees of freedom. ns is the natural cubic spline function. Time is the time variable. Dow is a categorical variable representing the day of the week effect. Holiday is a binary categorical variable to represent the holiday effect.

Previous studies have demonstrated that the lag effect of air pollution on disease does not extend beyond 7 days [26,27]. Therefore, when the effect of NO_2_ on CKD hospitalization was analyzed, the results were calculated from the same day (lag0) to a lag of 7 d (lag1~lag7), cumulative same day and first 1 d (lag0-1, i.e., cumulative 1 d) to the same day and first 7 d (lag0-7, i.e., cumulative 7 d), respectively.

In addition, subgroup analyses were used to explore population susceptibility. The main categories were gender (male and female), age (<65 years and ≥65 years), and season (April to September for the warm season and October to March for the cold season).

Finally, we performed a sensitivity analysis. We selected the day with the strongest effect of NO_2_ on CKD hospitalization and, based on the single-pollutant model, constructed a two-pollutant model by incorporating other co-existing pollutants. Additionally, we varied the degrees of freedom for the time variables (df = 6, 7, 8, 9) and assessed the robustness of the model by observing whether the effect estimates changed.

In this study, the effect of short-term exposure to NO_2_ on CKD hospitalization was quantified using the relative risk (RR) and the corresponding 95% confidence interval (CI) for each 10 µg/m^3^ increase in NO_2_ concentration. Statistical analyses were performed using the “dlnm” and “mgcv” packages in R 4.2.3 software. Plots were generated using the Origin 2024 software.

## 3. Results

Table 1 demonstrates the hospitalization of CKD patients during the study period. A total of 35,857 CKD patient hospitalizations were recorded. The number of males was 21,655 with a percentage of 60.39%. The number of females was 14,202 with a percentage of 39.61%. The number of patients aged <65 years was 28,303 with a percentage of 78.93%. The number of persons aged ≥65 years was 7554 with a percentage of 21.67%.

Table 2 presents the general characteristics of air pollutants and meteorological factors during the study period. The average NO_2_ concentration was 47.36 µg/m^3^, with a range of 7.80 to 146.60 µg/m^3^. The mean daily temperature was 11.34 °C, and the average relative humidity was 51.03%.

Table 3 presents Spearman’s correlations between air pollutants and meteorological factors. Moderate positive correlations were observed between PM_2.5_, PM_10_, SO_2_, CO, and NO_2_. In contrast, weak negative correlations were found between O_3_, temperature, relative humidity, and NO_2_. The correlation coefficient between temperature and relative humidity was −0.01. Additionally, PM_2.5_ and PM_10_, as well as CO and SO_2_, showed strong positive correlations.

**Figure 1:**
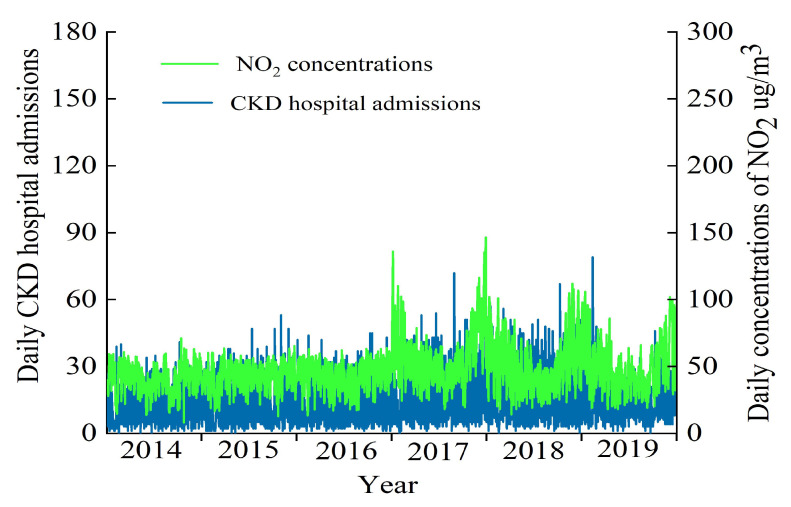
The change in NO_2_ daily mean concentration and CKD daily hospitalizations from 2014 to 2019 in Lanzhou, China.

Figure 1 illustrates the temporal variation in NO_2_ concentrations and CKD hospitalizations during the study period. NO_2_ concentrations exhibited a general pattern of higher levels in summer and fall, with lower levels observed during the off-season. The number of CKD hospitalizations demonstrated a consistent upward trend over the years.

**Figure 2:**
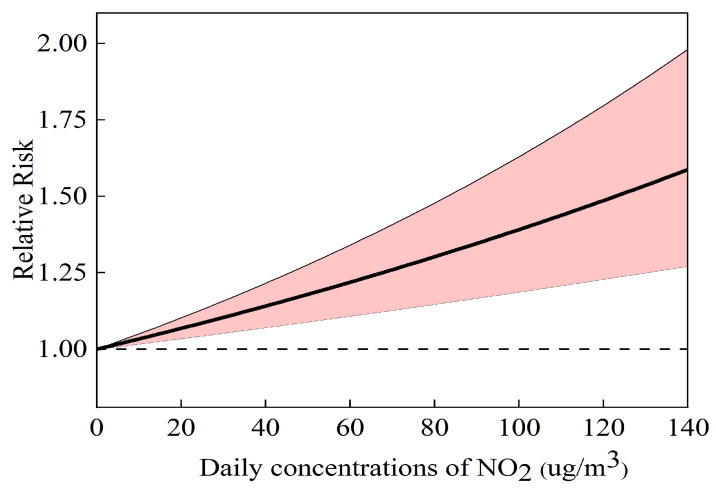
The exposure–response curves between NO_2_ and hospital admissions for CKD (lag0-1). The dashed line indicates the estimated relative risk, RR = 1. The solid line indicates the actual estimate of the risk of hospitalization for CKD from air pollutants. The shaded area indicates the 95% CI of the RR.

Figure 2 illustrates the exposure response (E-R) relationship between changes in NO_2_ concentration and the relative risk of CKD hospitalization at the time of maximum lag effect (lag0-1). The results show that the relative risk of CKD hospitalization increases as the NO_2_ concentration rises.

Table 4 presents the effect of changes in NO_2_ concentration on CKD hospitalization during the study period, along with the results of subgroup analyses. At lag0 and lag0-1 to lag0-4, NO_2_ concentrations were associated with an increased risk of CKD hospitalization. The maximum effect was observed at lag0-1 (RR = 1.034; 95% CI: 1.017, 1.050). In males with CKD, the NO_2_ exposure at lag0 and lag0-1 to lag0-4 also showed a harmful effect on hospitalization, with the largest effect at lag0-1 (RR = 1.030; 95% CI: 1.012, 1.048). In females, NO_2_ concentrations at lag1 and lag0-1 to lag0-7 had a deleterious impact on CKD hospitalization, with the maximum effect again observed at lag0-1 (RR = 1.039; 95% CI: 1.020, 1.060). For individuals aged < 65 years, NO_2_ exposure at lag0 and lag0-1 to lag0-4 was associated with a higher risk of CKD hospitalization, with the largest effect occurring at lag0-1 (RR = 1.034; 95% CI: 1.016, 1.051). In contrast, for individuals aged ≥65 years, NO_2_ exposure at lag7, lag0-1 to lag0-3, and lag0-7 was linked to an increased risk, with the maximum effect at lag0-7 (RR = 1.039; 95% CI: 1.008, 1.071). In the warm season, NO_2_ shows harmful effects only at lag 6 (RR = 1.011; 95% CI: 1.001, 1.022). In contrast, during the cold season, NO_2_ exhibits significant harmful effects across lag0-1 to lag0-6, with the most pronounced effect observed at lag 0–4 (RR = 1.047; 95% CI: 1.021, 1.074). Compared to the warm season, the harmful effects of NO_2_ are more pronounced in the cold season.

Table 5 presents the results of the two-pollutant model. Based on the findings from the single-pollutant model, we selected the maximum lag effect (lag0-1) and incorporated additional air pollutants into the two-pollutant model. The results indicate that the adjusted relative risk was higher compared to the single-pollutant model; however, the overall change was not statistically significant. This suggests that the model demonstrates robustness.

Table 6 presents the results of the sensitivity analysis. At lag0-1, the strongest lag effect, we examined the relationship between NO_2_ and CKD hospitalization by varying the degrees of freedom for the time variables (df = 6, 7, 8, 9). The results revealed minimal variation in RR (95% CI), suggesting that the model is stable.

## 4. Discussion

The present study employed the DLNM approach to investigate the short-term effects of changes in NO_2_ concentration on CKD hospitalization in Lanzhou, China, from 2014 to 2019. After the confounding effects of meteorological factors were adjusted for, the results demonstrated that short-term exposure to NO_2_ was associated with an increased risk of CKD hospitalization. Specifically, for each 10 µg/m^3^ increase in NO_2_ concentration, the relative risk of CKD hospitalization at lag0-1 was 1.034 (95% CI: 1.017, 1.050). A significant E-R relationship was observed between NO_2_ concentration and CKD hospitalization. Subgroup analyses further revealed that the effect of NO_2_ on CKD hospitalization was more pronounced in females and older adults. Additionally, the harmful effect of NO_2_ was more pronounced during the cold season.

The mechanisms underlying the effects of air pollution on CKD remain unclear. Potential pathways include elevated blood pressure, increased oxidative stress, enhanced inflammatory response, DNA damage, and metabolic disturbances [28]. Gaseous air pollutants, especially NO_2_, are predominantly generated from the combustion of fossil fuels. NO_2_ is easily inhaled into the lungs via the respiratory tract, where it can subsequently enter the bloodstream and exert potential adverse effects on kidney health [29]. Animal studies have shown that exposure to air pollutants can lead to a decrease in glomerular filtration rate, impaired urine concentration, and damage to the renal tubules in mice [30]. Collectively, these factors contribute to functional impairment of the kidneys, indicating that air pollution may pose a potential threat to kidney health.

The E-R curve is a crucial tool for public health risk assessment, offering a more visual representation of risk. In this study, we constructed the E-R curve for NO_2_ concentration and CKD hospitalization. The results revealed that higher NO_2_ concentrations are associated with a nonlinear increase in the relative risk of CKD hospitalization, characterized by a steep slope and the absence of a threshold effect. This finding is consistent with the E-R curves observed in previous related studies [21]. The construction of E-R curves helps elucidate the potential adverse health effects of air pollutants. It is imperative for local governments to implement measures aimed at improving air quality in order to safeguard public health.

The present study found a detrimental effect of NO_2_ exposure on CKD hospitalization, which is consistent with previous findings. A review of 13 studies reported an odds ratio (OR) of 1.10 (95% CI: 1.03, 1.17) for the risk of CKD associated with each 10 ppb increase in NO_2_ concentration [31]. A Korean study found that long-term exposure to NO_2_ was associated with an increased risk of death from end-stage renal disease, with a hazard ratio (HR) of 1.45 (95% CI: 1.09, 1.94) for each interquartile range (IQR) increase in NO_2_ concentration over a 7-year period [32]. A national study in China found that, for each 10 µg/m^3^ increase in NO_2_, the OR for CKD prevalence was 1.12 (95% CI: 1.09, 1.15) [33]. A meta-analysis of 32 studies showed that, for each 10 µg/m^3^ increase in NO_2_, the OR for CKD was 1.07 (95% CI: 1.05, 1.09) [34]. A European study found that, for each IQR increase in NO_2_ concentration, the HR for the risk of developing CKD was 1.06 (95% CI: 1.04, 1.08) [35]. These studies have consistently demonstrated that NO_2_ exposure has a deleterious effect on CKD. However, the magnitude of the effect estimates varies, which can be attributed to several factors, including regional differences in NO_2_ concentrations. Differences in population characteristics across regions, such as economic status and behavioral factors, are also associated with CKD [36]. In addition, the choice of study models can influence the results. For instance, some studies employed a generalized summation model, while others utilized DLNM.

Subgroup analyses by gender and age were conducted, revealing that the harmful effects of NO_2_ were more pronounced in females. A study conducted also found that NO_2_ had a more pronounced effect on female patients with glomerulonephritis in Hefei, China [37]. This may be attributable to differences in body structure. Our study results indicated that NO_2_ had a more pronounced harmful effect on individuals aged ≥65 years. A Korean study also reported a stronger effect of NO_2_ on mortality in CKD patients aged ≥65 years [38]. Potential causes of CKD include diabetes and hypertension [39]. These diseases are not only directly linked to renal functional decline through their pathogenesis but also indirectly exacerbate the renal burden by affecting systemic vascular health and metabolic status. For instance, diabetes induces a hyperglycemic state that damages both the renal tubules and glomeruli [40]. Hypertension and CKD can interact, further compromising renal function and posing a significant risk to overall health [41]. Additionally, aging is associated with declining physiological functions, and older individuals are often more exposed to environmental stressors such as air pollution. As a result, elderly CKD patients may be more susceptible to variations in NO_2_ concentration. This study also performed seasonal subgroup analysis, revealing a stronger effect of NO_2_ on CKD-related hospitalization during the cold season. It was consistent with previous study [21]. Lanzhou is a typical river-valley city surrounded by mountains, where the diffusion of air pollutants is often hindered. In winter, the climate is dry, with limited rainfall and an increased frequency of stagnant wind conditions. Furthermore, the extensive use of fossil fuels during the heating season results in higher emissions of air pollutants, exacerbating the pollution levels. Additionally, lower physical activity levels during winter contribute to a decline in immune function, making individuals more susceptible to the adverse health effects of air pollution. This study also identified harmful effects of NO_2_ during the warm season. Previous research has demonstrated that heat exposure has detrimental impacts on various health conditions, including kidney diseases [42,43]. During the warm season, the urban heat island effect may cause NO_2_ concentrations in urban areas to remain elevated. Furthermore, high temperatures facilitate the formation of secondary pollutants. These factors could be potential contributors to the observed harmful effects during the warm season.

This study examined the impact of changes in NO_2_ concentration on CKD hospitalization in Lanzhou, China. It not only addresses a research gap in this region but also contributes to the growing body of evidence on the harmful effects of NO_2_ on CKD. However, there are several limitations to this study. First, factors such as smoking, occupation, time spent outdoors, lifestyle, and personal characteristics (e.g., education and socio-economic status) were not included for control analysis, which may have influenced the results to some extent. Second, the use of data from monitoring stations as a proxy for population exposure levels introduces the potential for bias, as individual exposure levels may differ. Traffic pollution, in particular, can affect personal exposure. Additionally, this study focused solely on a single city, and no multi-city or national analysis was conducted. As a result, the findings may not be directly applicable to other regions.

## 5. Conclusions

Short-term exposure to NO_2_ has a detrimental effect on CKD hospitalization, with stronger associations observed in females and individuals aged ≥65 years. The harmful impact of NO_2_ is more pronounced during the cold season. In order to protect public health, effective measures should be implemented to reduce air pollution.

## Figures and Tables

**Table 1:** The number of CKD hospitalizations from 2014 to 2019 in Lanzhou, China.

	Number	Percentage (%)
**Total**	35,857	100
**Gender**		
Male	21,655	60.39
Female	14,202	39.61
**Age**		
<65 years	28,303	78.93
≥65 years	7554	21.07

**Table 2:** The general characteristics of air pollutants and meteorological factors from 2014 to 2019 in Lanzhou, China.

	Mean	SD	Min	25th	50th	75th	Max
**Air pollutants**							
PM_2.5_ (µg/m^3^)	48.97	26.91	9.00	31.34	42.57	59.26	278.00
PM_10_ (µg/m^3^)	114.85	82.91	16.00	71.00	99.52	136.82	1484.54
SO_2_ (µg/m^3^)	21.14	13.85	3.54	10.35	17.00	28.26	81.87
NO_2_ (µg/m^3^)	47.33	17.27	7.80	36.03	45.91	54.63	146.60
CO (mg/m^3^)	1.24	0.71	0.20	0.76	1.00	1.52	4.72
O_3_ 8 h (µg/m^3^)	88.24	38.77	8.00	58.00	82.00	114.00	222.00
**Meteorological factors**							
Temperature (°C)	11.34	9.83	−12.30	2.40	12.70	19.90	30.40
Relative humidity (%)	51.03	15.08	11.71	39.50	51.17	62.00	96.09

**Table 3:** The Spearman correlation between air pollutants and meteorological factors.

	PM_2.5_	PM_10_	SO_2_	NO_2_	CO	O_3_ 8 h	Temperature
PM_2.5_	1.00						
PM_10_	0.86	1.00					
SO_2_	0.66	0.58	1.00				
NO_2_	0.46	0.44	0.50	1.00			
CO	0.69	0.32	0.86	0.57	1.00		
O_3_ 8 h	−0.37	−0.15	−0.45	−0.07	−0.46	1.00	
Temperature	−0.51	−0.37	−0.64	−0.28	−0.58	0.62	1.00
Relative humidity	−0.14	−0.38	−0.25	−0.17	−0.02	−0.30	−0.01

**Table 4:** Relative risk (95% CI) of single-pollutant model and stratification analysis results in hospital admissions with CKD associated with a 10 µg/m^3^ increase in NO_2_ concentrations with different lag days.

Lag	Single Pollution Model	Subgroup Analysis					
		Gender		Age		Season	
		Male	Female	<65 Years	≥65 Years	Warm Season	Cold Season
0	1.019 (1.003, 1.034)	1.019 (1.003, 1.036)	1.018 (0.999, 1.037)	1.019 (1.003, 1.036)	1.016 (0.995, 1.038)	1.009 (0.981, 1.038)	1.010 (0.989, 1.031)
1	1.015 (0.998, 1.031)	1.010 (0.992, 1.028)	1.021 (1.001, 1.042)	1.014 (0.996, 1.032)	1.017 (0.993, 1.041)	1.005 (0.976, 1.035)	1.014 (0.991, 1.038)
2	0.998 (0.987, 1.010)	0.999 (0.987, 1.011)	0.998 (0.985, 1.012)	0.997 (0.986, 1.009)	1.003 (0.987, 1.018)	0.981 (0.960, 1.003)	1.013 (0.997, 1.030)
3	0.994 (0.987, 1.002)	0.994 (0.986, 1.002)	0.995 (0.986, 1.004)	0.995 (0.987, 1.002)	0.993 (0.983, 1.004)	0.985 (0.971, 1.000)	1.008 (0.997, 1.019)
4	0.995 (0.988, 1.003)	0.993 (0.985, 1.001)	0.998 (0.989, 1.007)	0.996 (0.988, 1.004)	0.991 (0.981, 1.002)	0.996 (0.981, 1.012)	1.001 (0.990, 1.012)
5	0.996 (0.989, 1.003)	0.995 (0.987, 1.002)	0.999 (0.991, 1.008)	0.997 (0.989, 1.004)	0.996 (0.986, 1.006)	1.005 (0.991, 1.019)	0.995 (0.984, 1.005)
6	0.998 (0.993, 1.003)	0.997 (0.992, 1.003)	0.999 (0.993, 1.005)	0.996 (0.991, 1.001)	1.005 (0.998, 1.013)	1.011 (1.001, 1.022)	0.989 (0.981, 0.996)
7	1.000 (0.989, 1.0106)	1.000 (0.989, 1.012)	0.997 (0.986, 1.012)	0.995 (0.984, 1.007)	1.017 (1.002, 1.032)	1.017 (0.997, 1.037)	0.982 (0.967, 0.997)
0-1	1.034 (1.017, 1.050)	1.030 (1.012, 1.048)	1.039 (1.020, 1.060)	1.034 (1.016, 1.051)	1.033 (1.011, 1.057)	1.014 (0.981, 1.049)	1.024 (1.001, 1.048)
0-2	1.032 (1.016, 1.048)	1.028 (1.011, 1.046)	1.038 (1.018, 1.057)	1.031 (1.014, 1.048)	1.036 (1.013, 1.059)	0.995 (0.961, 1.032)	1.038 (1.015, 1.062)
0-3	1.026 (1.008, 1.044)	1.022 (1.003, 1.041)	1.033 (1.011, 1.055)	1.026 (1.007, 1.045)	1.029 (1.004, 1.055)	0.981 (0.943, 1.020)	1.046 (1.020, 1.072)
0-4	1.021 (1.003, 1.010)	1.015 (0.996, 1.035)	1.031 (1.008, 1.053)	1.022 (1.002, 1.041)	1.020 (0.995, 1.047)	0.977 (0.937, 1.019)	1.047 (1.021, 1.074)
0-5	1.018 (0.998, 1.038)	1.010 (0.988, 1.031)	1.030 (1.005, 1.054)	1.018 (0.997, 1.040)	1.016 (0.988, 1.045)	0.982 (0.939, 1.027)	1.042 (1.014, 1.071)
0-6	1.016 (0.995, 1.037)	1.007 (0.985, 1.029)	1.029 (1.004, 1.054)	1.014 (0.992, 1.036)	1.022 (0.993, 1.052)	0.993 (0.948, 1.041)	1.030 (1.001, 1.060)
0-7	1.015 (0.994, 1.037)	1.007 (0.984, 1.031)	1.027 (1.001, 1.054)	1.009 (0.987, 1.032)	1.039 (1.008, 1.071)	1.010 (0.962, 1.060)	1.012 (0.982, 1.042)

**Table 5:** Relative risk (95% CI) in daily hospitalization of CKD with per 10 µg/m^3^ (lag0-1) increase in NO_2_ concentration in two-pollutant models.

	Two-Pollutant Models	Relative Risk (95% CI)
NO_2_	-	1.034 (1.017, 1.050)
	+PM_2.5_	1.034 (1.017, 1.052)
	+PM_10_	1.046 (1.022, 1.071)
	+SO_2_	1.046 (1.022, 1.068)
	+CO	1.045 (1.022, 1.068)
	+O_3_ 8 h	1.035 (1.018, 1.053)

**Table 6:** Relative risk (95% CI) for effects of NO_2_ on the stability of the model under varying degrees of freedom for time variable.

Degrees of Freedom	Relative Risk (95% CI)
6	1.032 (1.016, 1.049)
7	1.034 (1.017, 1.050)
8	1.033 (1.017, 1.050)
9	1.032 (1.016, 1.049)
10	1.034 (1.018, 1.051)

## Data Availability

The authors do not have permission to share the study’s data. However, it is available from the corresponding author.
